# *Pseudomonas syringae* on Plants in Iceland Has Likely Evolved for Several Million Years Outside the Reach of Processes That Mix This Bacterial Complex across Earth’s Temperate Zones

**DOI:** 10.3390/pathogens11030357

**Published:** 2022-03-15

**Authors:** Cindy E. Morris, Natalia Ramirez, Odile Berge, Christelle Lacroix, Cécile Monteil, Charlotte Chandeysson, Caroline Guilbaud, Anett Blischke, Margrét Auður Sigurbjörnsdóttir, Oddur Þ. Vilhelmsson

**Affiliations:** 1 INRAE, Pathologie Végétale, F-84140 Montfavet, France; odile.berge@inrae.fr (O.B.); christelle.lacroix@inrae.fr (C.L.); cecile.monteil@inrae.fr (C.M.); charlotte.chandeysson@inrae.fr (C.C.); caroline.guilbaud@inrae.fr (C.G.); 2 Faculty of Natural Resource Sciences, University of Akureyri, 600 Akureyri, Iceland; nataliar@unak.is (N.R.); mas@unak.is (M.A.S.); oddurv@unak.is (O.Þ.V.); 3 ÍSOR, Iceland GeoSurvey, Rangárvöllum við Hlíðarfjallsveg, 600 Akureyri, Iceland; anett.blischke@isor.is

**Keywords:** time tree, phyllosphere, microbial ecology, evolutionary history

## Abstract

Here we report, for the first time, the occurrence of the bacteria from the species complex *Pseudomonas syringae* in Iceland. We isolated this bacterium from 35 of the 38 samples of angiosperms, moss, ferns and leaf litter collected across the island from five habitat categories (boreal heath, forest, subalpine and glacial scrub, grazed pasture, lava field). The culturable populations of *P. syringae* on these plants varied in size across 6 orders of magnitude, were as dense as 10^7^ cfu g^−1^ and were composed of strains in phylogroups 1, 2, 4, 6, 7, 10 and 13. *P. syringae* densities were significantly greatest on monocots compared to those on dicots and mosses and were about two orders of magnitude greater in grazed pastures compared to all other habitats. The phylogenetic diversity of 609 strains of *P. syringae* from Iceland was compared to that of 933 reference strains of *P. syringae* from crops and environmental reservoirs collected from 27 other countries based on a 343 bp sequence of the citrate synthase (*cts*) housekeeping gene. Whereas there were examples of identical *cts* sequences across multiple countries and continents among the reference strains indicating mixing among these countries and continents, the Icelandic strains grouped into monophyletic lineages that were unique compared to all of the reference strains. Based on estimates of the time of divergence of the Icelandic genetic lineages of *P. syringae*, the geological, botanical and land use history of Iceland, and atmospheric circulation patterns, we propose scenarios whereby it would be feasible for *P. syringae* to have evolved outside the reach of processes that tend to mix this bacterial complex across the planet elsewhere.

## 1. Introduction

Strains of bacteria in the *Pseudomonas syringae* complex are found in association with a wide range of biological and inert substrates all over the world [1]. A remarkable feature of the *P. syringae* metapopulation is the worldwide ubiquity of certain genetic lines [2], and in particular, quasi-clones at the whole genome level isolated from locations as distant as France and New Zealand [3]. There is considerable evidence to support that this global mixing of *P. syringae* occurs mostly via the water cycle whereby cells of this group of bacteria are swept up into the atmosphere, travel long distances in the free troposphere, and fall back to Earth’s surface with rain and snow [1,2,4]. Nevertheless, the prevalence of agriculture in or near the regions from which all known strains of *P. syringae* have been isolated creates doubt about the relative importance of spill-over—from agriculture to natural habitats—by strains that could have been disseminated with exchanges of crop plant material. For strains in *P. syringae* phylogroups (PG) that have rarely been reported to be associated with cultivated plants, such as those in PG10 and PG13 for example [5], their world-wide prevalence is probably not due to exchanges of plant material but rather due to natural dissemination processes. However, for strains in PG01 and PG02, for example, that are associated with diseases of many cultivated plants [5], exchanges of plant material could contribute to worldwide movement, but to an unknown extent relative to that due to natural dissemination.

Iceland is ideal for exploring the natural biogeography of *P. syringae* given the small fraction of land dedicated to crop production. Only 1% of the total 10^5^ km^2^ of land surface of Iceland consists of arable land that is farmed whereas vegetation covers about 25% of the total land surface [6]. Average pesticide use in Iceland is about 20 g/ha of cropland due to the low level of insect pests in particular, leading to this country’s ranking as one of the lowest consumers of pesticides among 160 countries (https://www.worldometers.info/food-agriculture/pesticides-by-country/; accessed on 23 December 2021). Therefore, the natural vegetation is exposed to little pesticide spillover. The spring and summer seasons are relatively cool and wet with maximum temperatures rarely exceeding about 25 °C. These conditions would be particularly conducive for growth of *P. syringae*. 

*P. syringae* has not yet been reported from Iceland. The natural history of Iceland’s biodiversity leaves room for speculation about its presence. Modern natural vegetation of Iceland consists of nearly 500 vascular plant species, most of European origin. The monocots are the most diverse among the vascular plant groups (145 of the 275 species of angiosperms). There are also about 600 species of bryophytes [6]. Although the oldest fossil records of plants in Iceland date back to about 15 million years ago [7], it is believed that nearly all of the modern flora arose from rather recent immigration of plants. Iceland was formed from rifting and the spreading of the sea floor as the East Greenland continental margin of the North American tectonic plate began to move away from the Scandinavian and the British Isles margin of the Eurasian plate about 55 million years ago (YA) [6,8]. In spite of the vegetation that colonized ancient Iceland, it is currently accepted that little of the original flora survived the Last Glacial Maximum [6] that occurred 21,000 YA covering Iceland, Greenland, Canada, the British Isles, and Scandinavia in the northern hemisphere [9]. Much of the modern indigenous plant flora of Iceland is considered to have immigrated to the island at the time of the late Weichselian deglaciation (at about 10,000 YA) [7] after the Last Glacial Maximum. Interestingly, 97% of the vascular plants native to Iceland have also been recorded in Norway and about 86% in the British Isles; 64% are also found in Greenland [6]. Precursors of this vegetation are likely to have been dispersed with drift ice, water currents and birds from European and Eurasian origins [6]. The contribution of coastal flora from Norwegian fjords is likely to have occurred around 10,000 YA during the glacial melt that was rapid with a massive input of freshwater and sediments into the ocean. This is believed to have created a gyre of water with reduced salinity that fostered spread of biota from southwestern Norway to northern Atlantic islands [10]. It seems likely that microbes would have accompanied these migrations. In contrast to the potential for ocean currents to disseminate plants carrying *P. syringae* and other microorganisms, aerial dissemination to the island might be more difficult. Over the past 8000 years, at least, Iceland has often been beyond the main flow of the jet stream [11]. The jet stream can move northward to 65°N latitude crossing the southern half of the island depending on the behavior of the North Atlantic Oscillation, whereas the northern half is mostly crossed by Arctic winds [11]. This suggests that much of Iceland could be rather isolated from the main processes of natural long distance migration of terrestrial organisms. 

The objective of this work was to determine if strains in the *P. syringae* complex are indeed present on vegetation in Iceland and if so, to assess their abundance, their affinity for different plant types and habitats, and their diversity relative to that of *P. syringae* worldwide. 

## 2. Results

### 2.1. P. syringae Is Ubiquitous and Abundant on Vegetation across Iceland

**Figure 1:**
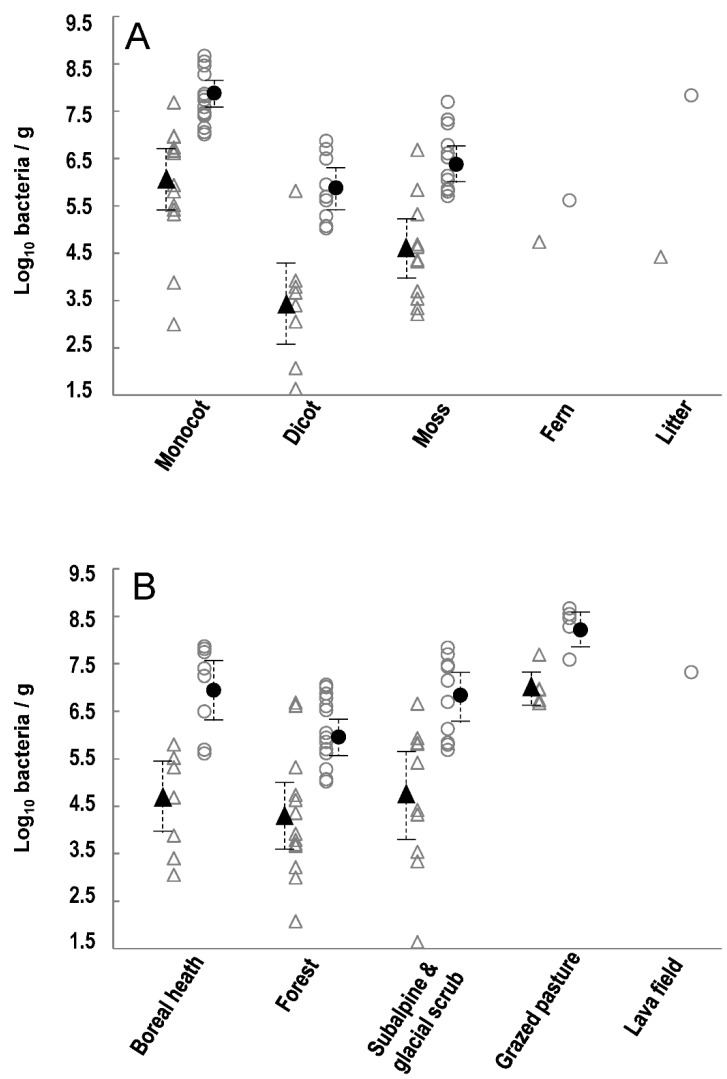
Population densities of *Pseudomonas syringae* (triangles) and of total mesophilic bacteria (circles) on plants collected across Iceland. Population sizes are presented according to (**A**) the type of plant and (**B**) the general habitat classification of the sampling site. Open symbols represent each of the 35 individual samples where population densities were greater than the detection level (ca. 40 cfu g^−1^). Closed symbols indicate the mean population densities and the dotted lines indicate the 95% confidence intervals.

Strains in the *P. syringae* complex were detected on 35 of the 38 samples collected at densities that ranged over six orders of magnitude from about 50 to 10^7^ cfu g^−1^. None of the plants showed any apparent disease symptoms. The analytical methods used could not distinguish if the isolated bacteria were on or in plant tissues. The total culturable mesophilic bacterial populations on these same plants ranged over ca. 3.5 orders of magnitude from 10^5^ to more than 10^8^ cfu g^−1^ (Table 1). Among the different plant types, *P. syringae* densities were significantly greatest on monocots compared to those on dicots and mosses (*p* < 0.05 for all significant comparisons) but there were no significant differences between these latter two plant types (Figure 1A). Densities of total mesophilic bacteria showed the same significant trends as those of *P. syringae* among the habitat types and plant types. Ferns, leaf litter and lava fields were each represented by only one sample therefore precluding statistical comparisons. There were significant effects of type of habitat from which samples were collected or whether the plant tissue was from monocots, dicots or moss on population densities of *P. syringae* and total culturable mesophilic bacteria (ANOVA, *p* < 0.05). According to Tukey’s HSD test, population densities of *P. syringae* were significantly greater in grazed pastures compared to all other habitats (*p* < 0.012 for all significant differences) whereas densities in the other habitats were not significantly different from each other (Figure 1B).

### 2.2. P. syringae in Iceland Is Genetically Diverse but Distinct from P. syringae Elsewhere

Assessment of the genetic diversity of strains was based on 343 bp of the citrate synthase (*cts*) gene. This segment of the *cts* gene aligned for a set of 609 *P. syringae* strains from Iceland and 933 strains of *P. syringae* from crops and environmental reservoirs collected from 27 countries on 8 continents or distinct geographical regions in North, South and Central America, Europe, UK, the Indian subcontinent, Africa, Asia, Australia and New Zealand. The *cts* sequences for the reference strains was obtained from our previous work [5] or from online sources (including https://www.ncbi.nlm.nih.gov/; accessed on 1 January 2020). This resulted in 259 haplotypes (i.e., unique *cts* sequences for the 343 bp) for the reference strains and 70 haplotypes for the Icelandic strains as listed in Supplementary Tables S1 and S2.

**Figure 2:**
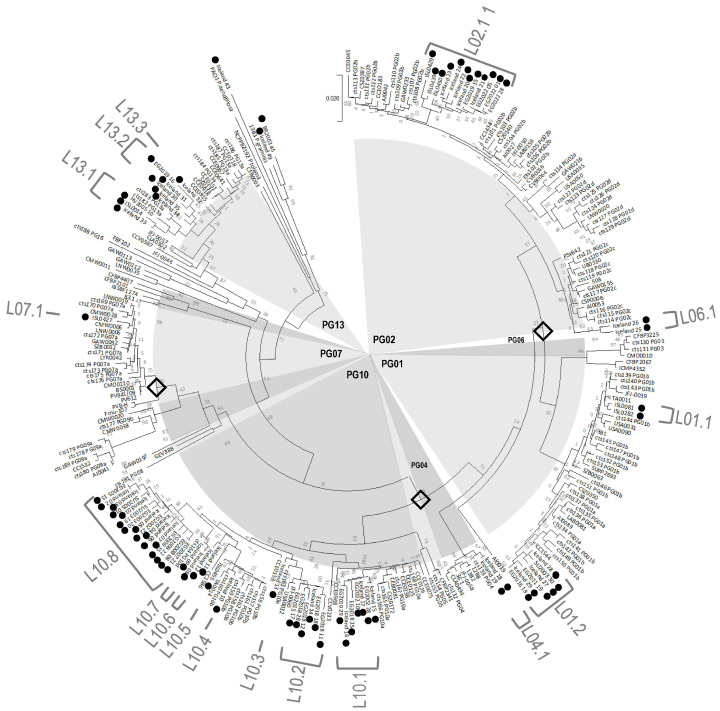
Phylogenetic situation of 329 haplotypes of *Pseudomonas syringae* representing 609 strains from Icelandic vegetation and 933 reference strains from 27 countries and a dozen types of substrates (crop plants, wild plants and various environmental substrates). The Neighbor-joining tree was constructed from a 343 bp sequence of the citrate synthase gene for one representative of each of the 329 haplotypes in the database based on 1000 bootstrap replications. The scale bar represents the p-distance. Haplotypes from Icelandic strains are indicated by black dots. Three additional Icelandic haplotypes and two reference haplotypes were included as outliers to root the tree. None of the Icelandic haplotypes included reference strains. Lineages (L) of Icelandic strains are denoted by grey bars and labels according to their phylogroup. Black diamonds on the tree branches indicate where time constraints were set to estimate the time of divergence of the Icelandic lineages (the divergence of the canonical group of *P. syringae* (PGs 1, 2, 3, 4 and 6) from the other phylogroups; the divergence of PG01 from PG02; and the divergence of PG07).

The haplotypes of *P. syringae* from Iceland were interspersed phylogenetically among the previously described diversity of this group of bacteria and represented PGs 1, 2, 4, 6, 7, 10, and 13 (Figure 2). Among the 70 haplotypes of strains from Iceland, none of them contained reference strains from outside of Iceland, illustrating an extreme extent of geographic isolation compared to the reference strains. In contrast, for reference strains there were 88 haplotypes containing more than one strain for which we assessed their geographic distribution. Of these haplotypes, 37 had strains from two or more continents representing 64% of the strains for these 88 haplotypes. The most widely dispersed case was one haplotype that contained strains from six continents or distinctly separated regions—Africa, Asia, the UK, North and Central America, continental Europe and Australia and New Zealand (Supplementary Figure S2A).

The distinction of the lineages did not seem to be related to their specialization to a type of plant or habitat. Many of the haplotypes were grouped into monophyletic lineages, of which seven lineages contained from 3 to 16 haplotypes (Figure 2). The largest of these lineages illustrated that diversification of these genetic groups of *P. syringae* were not specific to any plant type or habitat. For example, strains in lineage L02.1, that contained 12 haplotypes, were isolated from monocots, dicots and moss in forests and grazed pastures. Likewise, strains in lineage L10.8, that contained 16 haplotypes, were isolated from monocots, dicots, moss and fern in all of the four habitat categories (Supplementary Figure S2B). Furthermore, strains from nine haplotypes across all of the lineages were isolated from at least two plant types.

**Figure 3:**
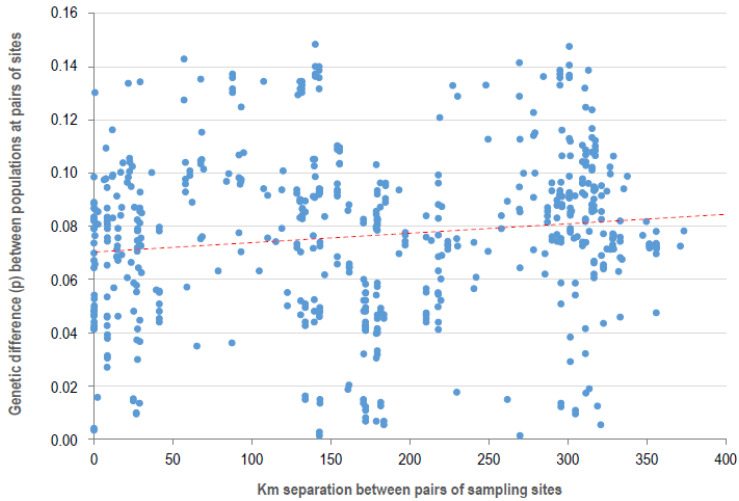
Effect of the geographic distance between sampling sites in Iceland on the genetic difference of the populations of *Pseudomonas syringae* they harbored. The genetic difference between sites was expressed as the percent dissimilarity of the 343 bp sequence of the citrate synthase gene used for phylogenetic analyses. The dotted line represents the regression curve: Genetic distance = 0.07134 + 0.00003123 × km.

We also evaluated if the genetic isolation of *P. syringae* in Iceland was due in part to limited mixing and dissemination of populations of this bacterium across the island. To accomplish this, we fitted a linear regression between mean genetic diversity and geographic distance for 531 pairwise comparisons among the 34 sites where more than one strain was isolated (Figure 3). This resulted in an R^2^ value for the regression between these two parameters of 0.014, indicating that only 1.4% of the variability in the difference in genetic diversity between sites is explained by geographic distance thus corroborating the hypothesis of mixing across the island. On the other hand, of the 70 haplotypes of *P. syringae* on Icelandic vegetation, 51 were found on only a single sample. Nevertheless, the most ubiquitous haplotype was in PG02 and was found on nine samples collected at diametrically opposed locations on the east and west coasts. Likewise, a haplotype of PG13 was detected on eight samples with a geographic distribution similar to that of the ubiquitous haplotype of PG02.

### 2.3. The Size, Composition and Co-Occurrence of Certain Phylogroups of P. syringae Populations on Vegetation Depend on Habitat and Plant Type

**Figure 4:**
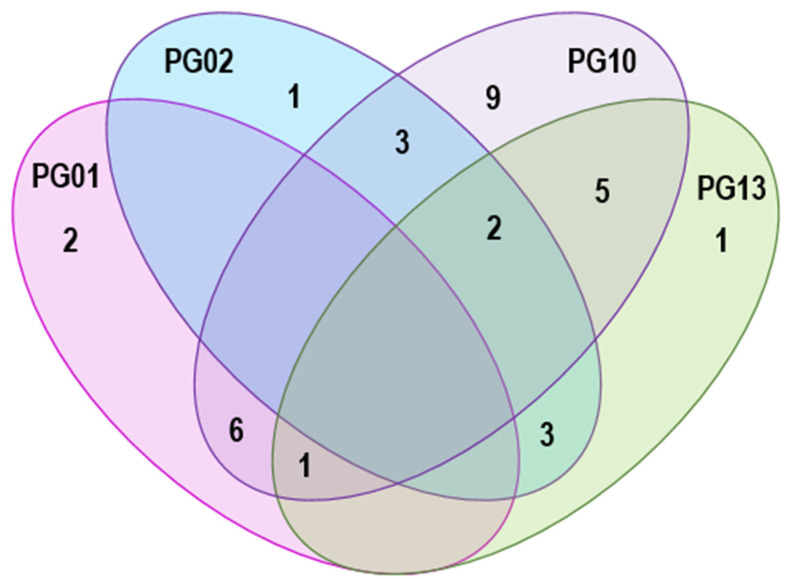
Venn diagram of the co-occurrence of strains of *Pseudomonas syringae* in phylogroups (PG) 1, 2, 10 and 13 in 33 samples of monocots, dicots and moss from Iceland. Integers represent the number of samples with co-occurrence of these phylogroups.

For the samples in which *P. syringae* was detected, PGs 1, 2, 10 and 13 were detected most frequently: on plant types that were sampled repeatedly (33 samples of angiosperms and moss), strains in PG10 were the most frequently detected (on 82% of the samples) followed by PG13 (36%) and PG01 and PG02 (each at 27%). PGs 4, 6 and 7 were detected in fewer than 6% of these samples. There was co-occurrence of different phylogroups in the same sample for about 60% of the samples, but, interestingly, PG01 and PG02 were the only phylogroups that did not occur together in any of the samples (Figure 4).

**Figure 5:**
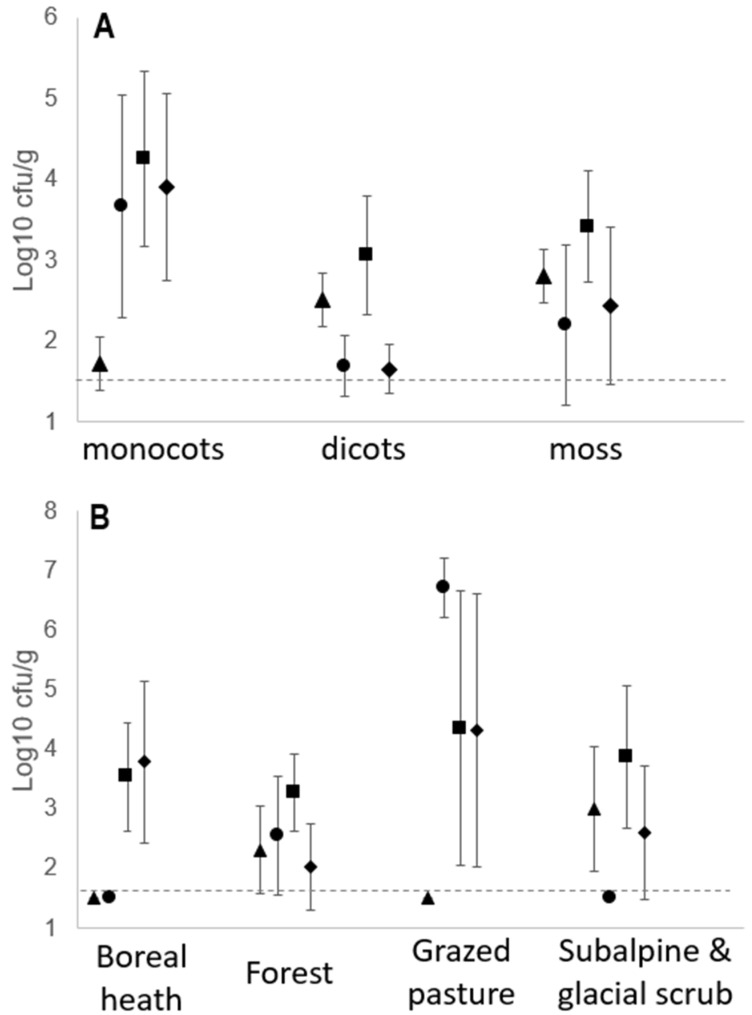
Population densities of the four phylogroups of of *Pseudomonas syringae* most frequently detected on vegetation in Iceland for different types of plants (**A**) and in different habitats (**B**). Mean population densities of PG01 (triangle), PG02 (circles), PG10 (squares) and PG13 (diamonds) were calculated based on results for all samples in each category. Values for samples with population sizes under the detection level were set to just below the detection limit (10^1.5^) rather than excluding the sample from the calculation. Error bars represent the 95% confidence interval. The dotted line represents the limit of detection of *P. syringae* in these samples.

Vegetation type (monocots, dicots or moss) had an effect on the abundance of certain phylogroups of *P. syringae*. Whereas monocots tended to harbor larger populations than dicots and mosses for most phylogroups (Figure 5A), populations of PG01 were virtually absent on monocots (detected on only one of 14 samples). Furthermore, PG10 had the largest populations of all phylogroups consistently for monocot, dicots and ferns (Figure 5A). On the one sample of fern collected (not shown in Figure 5), PG10 constituted 100% of the 5.4 × 10^4^ cells of *P. syringae* detected. However, the differences in population size of the phylogroups among plant types was statistically significant only for PG13 (ANOVA, *p* = 0.017).

Across the different habitats sampled, only PG10 and PG13 were consistently present in all habitats. Neither PG01 nor PG02 were detected in any of the seven Boreal heath samples. In addition, PG01 was not detected in the five grazed pasture samples, and PG02 was not detected in any of the ten samples from subalpine and glacial scrub. Among all phylogroups, PG02 was the only one to be significantly influenced by habitat, having the significantly greatest abundance in samples from grazed pastures.

### 2.4. Haplotypes of P. syringae on Icelandic Vegetation Diverged near the Time of the First Fossil Evidence of Plants on Iceland as Well as Very Recently

**Figure 6:**
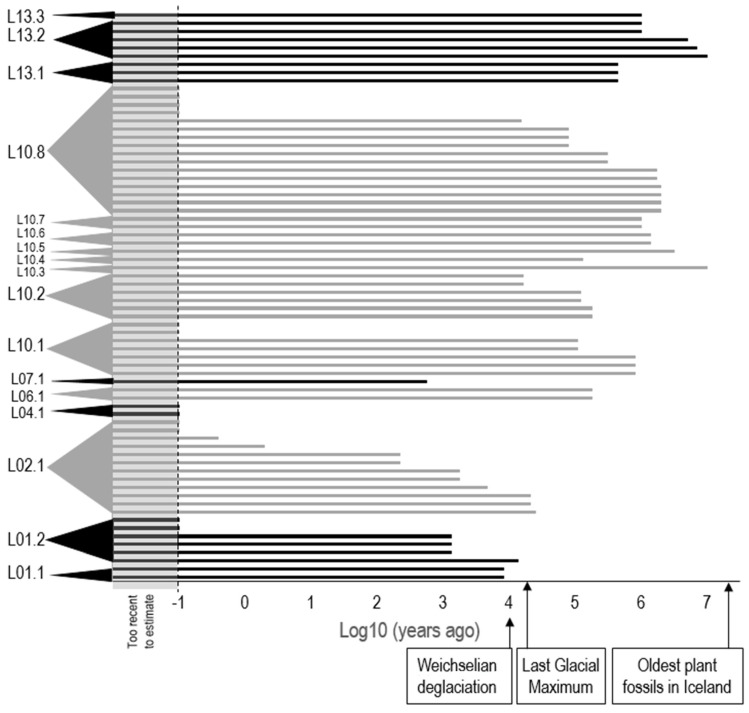
Estimated time of divergence of the 70 haplotypes of *Pseudomonas syringae* isolated from plants in Iceland. Each bar represents a haplotype. Haplotypes are grouped according to their lineages (L) in phylogroups 1 to 13 as indicated in Figure 2. Times of divergence, presented on a log scale, ranged from 16 million years ago to too recent to estimate (less than the value indicated by the dotted line). Each successive phylogroup is presented by either black or grey bars to help the reader differentiate among adjacent phylogroups in the figure.

The estimated time of divergence of the 70 *cts* haplotypes of *P. syringae* from Icelandic vegetation ranged from 16 million years ago to too recent to calculate (Figure 6). The time of divergence of all haplotypes in PGs 1, 2, 4 and 7 was estimated to have occurred at the time of, or after, the Last Glacial Maximum. This estimate is compatible with a process of colonization and evolution driven by the modern flora after the period of deglaciation. In contrast, the estimated time of divergence of all haplotypes of PGs 6 and 13 and most of PG10 are markedly older than the last glacial maximum.

## 3. Discussion

Understanding how plant pathogens evolve is essential for anticipating disease emergence and the durability of resistant plant varieties. Overwhelmingly, knowledge of the evolution of plant pathogens has been founded mostly on studies of the specific crop-pathogen interactions of interest in a context of modern agricultural practices and settings. Such studies provide only partial information, ignoring the consequences of stresses and selective pressures that plant pathogens could endure in reservoirs and natural habitats. This biases our understanding of evolutionary processes that microorganisms with pathogenic capacities endure. The lack of insight on stresses and selective pressures other than in agricultural contexts inevitably biases the importance accorded to agriculture in the evolution of plant pathogens. Over the past several decades there have been efforts to disentangle anthropogenic forces from non-anthropogenic forces in the evolution and emergence of zoonotic pathogens and parasites [12,13,14] leading to the establishment of comprehensive surveillance systems under the concept of One Health [15]. However, for plant pathogens it is legitimate to wonder whether it is possible to find contexts in which to study the influence of non-agricultural habitats on pathogen evolution. The preponderance of crops in the planetary landscape and the influence of long distance dissemination that can move microorganisms between natural and agricultural contexts make it difficult to find such contexts. In a previous study we determined that more than half of the haplotypes of *P. syringae* in headwaters of rivers were endemic to the site where they were isolated (in either France, the US or New Zealand) [2]. However, the possible influence of agriculture and aerial dissemination on those headwater sites make it difficult to assess if these haplotypes diverged mostly under the influence of their aquatic habitat or if they were refugees from agriculture.

Our results illustrate that the geographical, geological and ecological contexts of Iceland create a remarkable situation where we can assess the influence of natural landscapes on the diversification of a group of bacteria that contain plant pathogens. We have shown that monocots, dicots, moss and ferns across four habitat types (boreal heath, forests, grazed pastures, subalpine and glacial scrub) all harbor *P. syringae*, but that grazed pastures in particular have the greatest influence on the fitness of *P. syringae*. Some of the pastures were sown monocultures but other were natural mixed stands. Strains in PG01 and PG02 occurred only in two of the four habitats, their frequencies of occurrence have opposing trends on monocots and dicots, and they seemed to exclude each other in the samples we analyzed. Interestingly, these two groups are represented by important plant pathogens in the overall metapopulation of *P. syringae* [5]. In contrast, strains in PG10 and PG13—phylogroups for which there are no reports of them causing plant disease outbreaks-occurred with roughly equal frequency and abundance across all the habitat types and plant types we examined. This suggests a greater adaptability of phylogroups 10 and 13 compared to PG01 and PG02. Nevertheless, as we noted for populations of *P. syringae* in headwaters in France, the US and New Zealand [2], we observed in Iceland that PG02 has the capacity to generate haplotypes that are more ubiquitous than haplotypes of other phylogroups. In other words, although representatives of PG02 are not necessarily the most frequently encountered in the environment compared to PG10 and PG13, when the occurrence of individual haplotypes are noted, those of PG02 are the more ubiquitous than those from other phylogroups. This suggests that the pangenomic traits that assure adaptability tend to be packed into individual haplotypes of PG02 more so than in the other phylogroups where these traits might tend to be distributed across individual haplotypes. This hypothesis could be explored by searching for particular genomic features of ubiquitous PG02 haplotypes compared to haplotypes that are endemic to various geographic locations.

We believe that it is possible to attribute the trends described above mostly to processes that do not directly involve crop cultivation. This is because cultivation of pastures and other crops is very recent on the evolutionary scale of *P. syringae* and a very small fraction of land is used for cultivation in Iceland. Furthermore, the composition of *P. syringae* populations in Iceland is apparently separate from the influence of immigrants from regions of the world where crop cultivation is much more predominant. By comparing Icelandic strains with a comprehensive data set of *P. syringae* strains from 27 countries and a dozen types of habitats, we revealed the uniqueness of Icelandic strains and the unlikelihood that some of them had emigrated recently from other cultivated regions of the globe. Indeed, this result is based on the data set of reference strains that we established. This data set is very comprehensive in terms of geographic and environmental origins of strains (Supplementary Table S1, Supplementary Figure S3A). Both data sets, for Icelandic and for reference strains, illustrate that for many of the haplotypes and genetic lineages of *P. syringae*, their corresponding strains are found in several habitats or geographic locations and in association with several types of substrates. This observation argues against the notion that Icelandic strains are specialized to a given habitat or plant type and it gives additional credence to our conclusion that there is an impediment to the long distance dissemination of *P. syringae* toward Iceland. Nevertheless, future samples that can expand on this diversity might lead to nuances in the interpretation of the results. In particular, our data set of reference strains does not contain any samples from latitudes greater than 64° whereas all of the Icelandic strains are from sites situated farther north than 64° which seems to be the limit of the reach of the jet stream in the Northern Hemisphere [11]. The geographic range of many of the plant species we sampled here, including *Alchemilla alpina*, *Empetrum nigrum*, *Vaccinium uliginosum*, and the mosses *Hylocomium splendens* and *Rhytidiadelphus loreus*, for example, includes expanses of North American, northern Europe and Greenland north of 64°. Assessing the diversity of *P. syringae* from these plants at different locations could contribute to validating the uniqueness of the Icelandic strains. That Iceland is an island separated by at least 300 km of ocean from the nearest neighboring land mass might exaggerate phenomena that could be occurring elsewhere at high latitudes. Experiments to capture *P. syringae* in rain from offshore winds along the eastern coast of Iceland, from where most rain events arrive, could further corroborate the limited access of Iceland via the atmosphere. Notwithstanding, the apparent seclusion of Icelandic populations of *P. syringae* is coherent with knowledge of the range of atmospheric circulation [11] that would have limited dissemination to the island from other continents where *P. syringae* is abundant. In this light we propose that the diversity of *P. syringae* from vegetation in Iceland is, in large part, a reflection of evolutionary processes occurring in habitats with very little or no crop cultivation. However, grazing—that involves regular wounding to plants (mostly grasses) and input of nitrogen-laden fecal matter by herbivores—enhances the fitness of most phylogroups of *P. syringae* with the notable exception of PG01. Grazing of pastures is a practice that started in Iceland in about the 900′s with the introduction of sheep, before which there were no herbivorous mammals on the island. A unique aspect of cold environments is that mosses, in addition to grasses, can also be grazed—by geese, sheep and reindeer [16]. Iceland presents the unique opportunity to determine if grazing of mosses has the same impact on populations of *P. syringae* as does grazing of grasses, and to evaluate the dynamics of this impact under the grass-moss-herbivore feedback that is forecast in a warming climate [17].

By estimating the time of divergence of the unique Icelandic lineages of *P. syringae*, we can propose a hypothetical scenario of the origin and evolution of *P. syringae* in Iceland in the context of the geological and botanical history of the island. The precision of our estimates of age could be improved if longer sequences (such as concatenated housekeeping genes or whole core genomes) were available. Nevertheless, the estimates we have made here allow us to propose a scenario that has not been told previously for a plant pathogen. The ages of the Icelandic lineages of PG01, PG02 and PG07 are compatible with the hypothesis that progenitors of these lineages arrived with plants that invaded and colonized Iceland just after the Last Glacial Maximum leading to today’s modern flora of the island mostly from Norway and partially from the northern reaches of present-day Scotland [6]. These immigrant lineages apparently diversified, thereby giving rise to the very young endemic lineages we detected for PG01 and PG02 (Figure 6). We suspect that a similar scenario of immigration after the Last Glacial Maximum, and then subsequent diversification of lineages, occurred for PG04 but that the older lineages were too rare to detect in our sampling or have died out.

In contrast to PGs 1, 2 and 7, the ages of the lineages of PG10 and PG13 suggest that they were present before, and survived, the Last Glacial Maximum. This is indeed what is believed to have occurred for certain microscopic freshwater crustaceans that have evolved unique genetic lines endemic to Iceland; they probably survived glaciation under the ice sheet [18]. There is evidence that bacteria could have survived glaciation in the ice sheet and/or in subglacial lakes. Concerning subglacial lakes, their temperature and chemical conditions can be very similar to mountain lakes and headwaters that harbor *P. syringae* [2]. The pH, temperature and conductivity of the subglacial lake in the Grímsvötn volcanic caldera in Iceland, for example, are within the range of those in freshwaters where *P. syringae* has been frequently detected [19]. The Grímsvötn volcanic caldera lake and its sediments harbor culturable, aerobic, psychrophilic bacteria at concentrations of ca. 6 × 10^6^ L^−1^ and 8 × 10^6^ g^−1^, respectively [19]. Bacterial populations in the water and sediments of this subglacial lake consist mostly of beta- and gamma-Proteobacteria with *Pseudomonas* spp. detected in the sediments [19]. To have survived within ice during the Last Glacial Maximum, *P. syringae* would have needed to endure, at most, the period that started about 33,000 YA when the ice sheet covering the North Atlantic started growing [20] to about 10,000 YA when rapid deglaciation occurred [7], i.e., a period of roughly 20,000 years. Although there is no direct evidence that *P. syringae* could survive this duration, diverse viable bacteria have been isolated from cores of 20,000 year-old ice [21]. Furthermore, metabolic activity has been demonstrated for bacteria in ice from Lake Vostoc that is 110,000 to 240,000 years old [22]. These observations illustrate the capacity of bacteria to survive for tens to hundreds of thousands of years in ice and they are the basis for concern about the diversity of microorganisms that are being liberated from melting permafrost and glaciers [23]. For *P. syringae*, its capacity to survive under annual snow pack near the snow-ground interface and its subsequent percolation into karsts and rivers upon melting of the snow in the spring have been demonstrated [24,25], but direct observations for longer periods have not been reported. Whatever the processes by which PG10 and PG13 survived the Last Glacial Maximum, the age of their lineages in Iceland suggest that they colonized the island via the same processes that brought the original flora to Iceland. For PG10, we observed that these original lineages diversified to give rise to the younger lineages we observed. For PG13, our samples did not lead us to observe subsequent diversification if it has occurred.

If *P. syringae* has had the opportunity to evolve in relative seclusion from the rest of the metapopulation of this bacterial complex elsewhere on Earth, it is tempting to wonder if other plant-associated microorganisms typically disseminated via the atmosphere have also evolved in similar seclusion. Comparative ecology between *P. syringae* and other broad-host-range, aerially disseminated pathogens, such as *Botrytis cinerea*, has led to elucidation of general ecological concepts applicable to numerous organisms and to the particularities of each organism [26,27]. Icelandic landscapes provide a unique opportunity to pursue comparative ecological studies on the relative impacts of agricultural and nonagricultural contexts on the diversification of plant pathogens.

## 4. Materials and Methods

### 4.1. Samples and Sampling Sites

**Figure 7:**
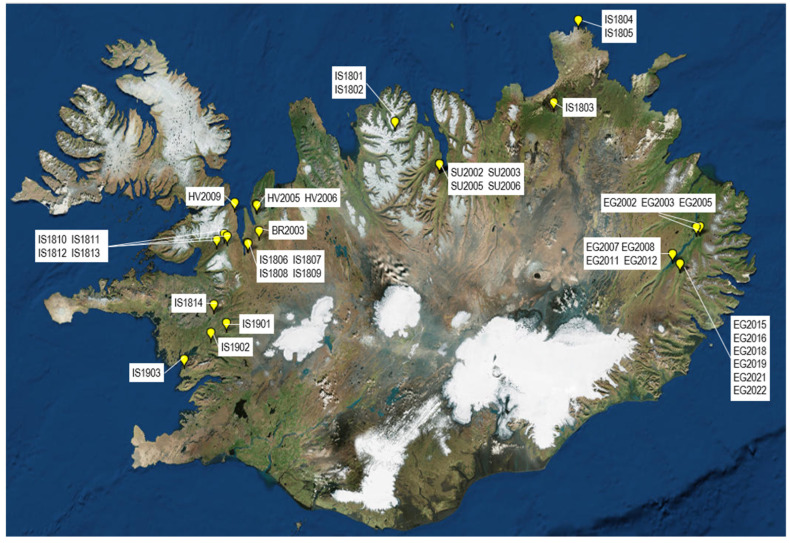
Location of the 38 sites where vegetation was sampled in 2018, 2019 and 2020 across Iceland. Codes for each site are the sample names indicated in Table 1. The map was made with tools at GPSVisualizer.com, accessed on 29 June 2021.

In the summer months of 2018, 2019 and 2020, 38 samples of plants (angiosperms, moss and ferns) (Table 1) were collected from 32 sites in the Western Northwestern, Northeastern and Eastern Regions of Iceland (i.e., all regions except the Westfjords, Reykjanes and the Southern Region) (Figure 7). The sampling sites were classed into habitat categories (boreal heath, forest, subalpine and glacial scrub, grazed pasture, lava field) based on classifications of the Icelandic Institute of Natural History (https://vistgerdakort.ni.is/; accessed on 29 June 2021), the European Nature Information System classifications for Iceland (https://en.ni.is/flora-funga/habitat-types/terrestrial-habitat-types; accessed on 29 June 2021) and on ground-based observations at each of the sampling sites.

Plant tissue was collected aseptically into clean, single-use plastic bags. Most plants were from natural stands of mixed species. For each sample, a quadrat of about 10 × 10 cm was delimited and all plants of a given species or type (dicot, monocot or moss) in the quadrat were collected separately. Single species were sampled separately where they could be identified, as was the case for dicots. Otherwise, plants in the same quadrat were bulked by recognizable groups such as monocots, dicots or moss. Samples consisted of a bulk of the plants sampled from three, randomly placed quadrats per each geographic site. They were stored in a cooler while transported to the laboratory.

**Table 1:** Population densities of *Pseudomonas syringae* and total culturable mesophilic bacteria on plant samples collected across Iceland. The locations where samples were collected are indicated in Figure 7 according to the sample names.

Sample Name	Date	Latitude °N	Longitude °W	Habitat Type	Category of Plant Sample	Identity of Sampled Material	log_10_ cfu *P. syringae*/g Tissue	log_10_ cfu Total Bacteria/g Tissue
BR2003	Augustust 2020	65.239038	−20.851623	Forest	monocot	mixed grasses	6.62	7.01
EG2002	September 2020	65.262654	−14.378452	Forest	moss	*Hylocomium splendens*	3.70	6.05
EG2003	August 2020	65.262654	−14.378453	Forest	monocot	*Festuca* sp.	2.99	7.06
EG2005	September 2020	65.267478	−14.331496	Forest	dicot	*Empetrum nigrum*	2.07	5.07
EG2007	September 2020	65.096122	−14.732982	Forest	moss	*Hylocomium splendens*	3.21	5.86
EG2008	September 2020	65.096122	−14.732983	Forest	fern	*Equisetum pratense*	4.74	5.62
EG2011	September 2020	65.094289	−14.734456	Forest	dicot	*Rubus saxatilis*	3.79	5.28
EG2012	September 2020	65.094289	−14.734457	Forest	moss	*Rhytidiadelphus loreus*	5.32	6.61
EG2015	August 2020	65.036883	−14.620112	Forest	dicot	*Salix arctica*	3.67	5.95
EG2016	September 2020	65.036883	−14.620113	Forest	moss	*Rhytidiadelphus squarrosus*	4.36	6.78
EG2018	September 2020	65.036937	−14.620176	Forest	dicot	*Vaccinium uliginosum*	3.92	5.03
EG2019	September 2020	65.036937	−14.620177	Forest	moss	*Rhytidiadelphus loreus*	4.63	6.52
EG2021	September 2020	65.036937	−14.620176	Forest	dicot	*Alchemilla alpina*	nd^1^	6.87
EG2022	September 2020	65.036937	−14.620177	Forest	moss	*Hylocomium splendens*	6.68	5.70
HV2005	August 2020	65.402968	−20.896696	Boreal heath	dicot	*Alchemilla alpina*	3.06	6.50
HV2006	August 2020	65.400877	−20.884433	Boreal heath	dicot	*Alchemilla alpina*	nd	5.62
HV2009	August 2020	65.411194	−21.215754	Boreal heath	dicot	*Salix herbacea*	3.40	5.69
IS1801	June 2018	65.904903	−18.838611	Subalpine & glacial scrub	monocot	sparse mixed grass	5.94	7.43
IS1802	June 2018	65.925386	−18.823611	Subalpine & glacial scrub	monocot	sparse mixed grass	6.66	7.47
IS1803	June 2018	66.024216	−16.493893	Subalpine & glacial scrub	litter	litter on forest floor	4.42	7.84
IS1804	June 2018	66.5145298	−16.132639	Subalpine & glacial scrub	monocot	mixed grasses and mosses	5.42	7.15
IS1805	June 2018	66.514528	−16.132639	Subalpine & glacial scrub	moss	mixed grasses and mosses	4.33	6.13
IS1806	June 2018	65.163587	−21.032756	Boreal heath	monocot	mixed grasses and mosses	5.52	7.81
IS1807	June 2018	65.163587	−21.032756	Boreal heath	moss	mixed grasses and mosses	3.88	7.40
IS1808	June 2018	65.162114	−21.019500	Boreal heath	monocot	mixed grasses and mosses	5.33	7.86
IS1809	June 2018	65.162114	−21.019500	Subalpine & glacial scrub	moss	mixed grasses and mosses	5.84	7.69
IS1810	June 2018	65.205417	−21.326132	Boreal heath	monocot	mixed grasses and mosses	5.80	7.74
IS1811	June 2018	65.205417	−21.326132	Boreal heath	moss	mixed grasses and mosses	4.68	7.24
IS1812	June 2018	65.218333	−21.370000	Grazed pasture	monocot	farm grass	6.97	8.54
IS1813	June 2018	65.184495	−21.480230	Grazed pasture	monocot	farm grass	6.74	8.28
IS1814	June 2018	64.777373	−21.520000	Lava field	moss	moss on lava rock	nd	7.32
IS1901	September 2019	64.660454	−21.338979	Grazed pasture	monocot	pasture grass and clover	7.69	8.67
IS1902	September 2019	64.601361	−21.563346	Grazed pasture	monocot	pasture grass	6.95	8.46
IS1903	September 2019	64.428018	−21.959702	Grazed pasture	monocot	pasture grass	6.67	7.58
SU2002	August 2020	65.649893	−18.183058	Subalpine & glacial scrub	dicot	Empetrum nigrum	5.82	6.70
SU2003	August 2020	65.649893	−18.183058	Subalpine & glacial scrub	moss	Hylocomium splendens	3.54	5.84
SU2005	August 2020	65.650320	−18.182976	Subalpine & glacial scrub	dicot	Empetrum nigrum	1.63	5.69
SU2006	August 2020	65.650320	−18.182977	Subalpine & glacial scrub	moss	Hylocomium splendens	3.34	5.80

### 4.2. Isolation, Characterization and Quantification of Bacteria

Samples were processed within 4 days of collection. A subsample (about 10 g) was weighed and stomached for 2–3 min in 0.1 M sterile phosphate buffer (8.75 g K_2_HPO_4_ and 6.75 g KH_2_PO_4_ per liter, pH 6.8) (5–10 mL buffer/g of tissue) in sterile stomacher bags. Macerations were dilution-plated on KBC medium [28] and 10% tryptic soy agar (TSA) as described previously [29] and incubated at room temperature (ca. 22–25 °C) in the dark. Colonies were counted after 2 and 3 days of incubation: total colonies on TSA and *P. syringae*-like colonies on KBC. Various colony-types were targeted as putative *P. syringae* to account for the morphological diversity of colonies across the *P. syringae* complex. The ultimate identity was then confirmed based on phylogenetic relationships with reference strains. *P. syringae*-like colonies are round with lace-like or irregular edges with various distinguishing features for some of the phylogroups (Supplementary Figure S1). For strains in all phylogroups except PG07 and PG08, colonies are about 0.5 mm in diameter with a surface that is smooth and translucent and not rough nor opaque. The colonies are not pigmented. Colonies of strains in PG07 and PG08 are up to 1 mm in diameter and slimy with a yellow/orange pigment. Many strains of *P. syringae* display phase variation where a round, smooth colony gives rise to a juxtaposed slimy colony. Most, but not all, strains of *P. syringae* produce fluorescent pigments on medium with low iron content; however, this is difficult to observe on KBC medium. Up to 30 *P. syringae*-like colonies were randomly chosen per sample and purified. Their identity as part of the *P. syringae* complex was verified based on the phylogenetic relationship of each strain with reference strains, established from partial sequences of the citrate synthase (*cts*) gene, according to the phylogenetic context, primers for *cts* amplification and conditions for PCR described by Berge and colleagues [5]. Amplified DNA was sent to GenoScreen (Lille, France) or Macrogen Europe B.V. (Amsterdam, The Netherlands) for sequencing. Values for population densities of *P. syringae* on plant tissues were calculated based on the percent of *P. syringae*-like colonies sampled from KBC that were verified to be part of the *P. syringae* complex among the total number of *P. syringae*-like colonies observed. A total of 736 strains from *P. syringae*-like colonies were characterized for which 609 were confirmed to be in the *P. syringae* complex. A list of all strains collected in Iceland, their origin and their associated cts sequence are presented in Supplementary Table S2.

Sizes of total and *P. syringae* populations were calculated as log10 colony-forming units (cfu) g^−1^ for each individual sample. Calculation of descriptive statistics (mean, standard error, confidence intervals) and comparative statistics (*p*-values from ANOVA) were calculated with Statistica 10 (StatSoft www.statsoft.fr; accessed on 27 August 2019).

### 4.3. Evaluation of Genetic Diversity and Divergence

Genetic diversity was assessed among Icelandic strains and in comparison to a data set of *cts* sequences from 933 reference strains that included those from our previous work [5] and from open access sources representing a dozen habitat types (wild and cultivated plants, surface freshwaters, irrigation water, ground water, lithic biofilms, leaf litter, cloud water, rain and snowfall, snowpack and soil) and 27 countries of isolation in the Northern and Southern hemispheres. The names, origins and associated cts sequences for these 933 reference strains are in Supplementary Table S1. Sequences were aligned and cut and then Neighbor-joining trees were constructed with MEGA 10.2.6 (https://www.megasoftware.net/; accessed on 26 June 2021) based on 1000 bootstrap replications as described previously [5]. Time of divergence of haplotypes from Iceland were approximated with the Timetree interface in MEGA 10.2.6 [30] using the maximum likelihood estimator with uniform distribution of age. Age constraints for calculation of divergence times were set according to previous estimates of the age of the most recent common ancestor of various phylogroups. The time of divergence of the canonical *P. syringae* phylogroups (PGs 1, 2, 3, 4, and 6) from the other phylogroups was set at 150–183 million years ago according to O’Brien and colleagues [31]. These same authors estimated the divergence of PG01 and PG02 at 3–10 million years ago. The age of divergence of PG07 was set at 0.3 million years ago according to estimates of Karasov and colleagues [32]. The specific placements of these time constraints on the phylogenetic tree are indicated in Figure 2.

## Data Availability

The data presented in this study are available in supplementary material.

## Supplementary Materials

The following supporting information can be downloaded at: https://www.mdpi.com/article/10.3390/pathogens11030357/s1, Figure S1: Morphology of Pseudomonas syringae colonies; Figure S2: Graphical representation of source categories of haplotypes of strains of Pseudomonas syringae from Iceland and from reference collections used in this study; Table S1: Data set of reference strains used in this study; Table S2: Data set for strains of Pseudomonas syringae isolated from vegetation in Iceland for this study [5,33,34,35,36].
