# Supplementary material for: Pseudomonas syringae on Plants in Iceland Has Likely Evolved for Several Million Years Outside the Reach of Processes That Mix This Bacterial Complex across Earth’s Temperate Zones
Source: Pathogens. 2022 Mar 15;11(3):357. doi: 10.3390/pathogens11030357 (PMC8951587; doi:10.3390/pathogens11030357)

**Supplemental Figure S1.** Morphology of *Pseudomonas syringae* colonies

Morris et al. *Pseudomonas syringae* on plants in Iceland has likely evolved for several million years outside the reach of processes that mix this bacterial complex across Earth's temperate zones. MDPI Pathogens 2022

Colonies of *Pseudomonas syringae* are smooth with translucent irregular edges. This is the phenotype for almost all phylogroups. In contrast some colonies in phylogroup 7 are mucoid but not fully opaque (as in the top right photo). Examples of colonies most similar to *P. syringae* produced by other species are presented in the bottom panel.

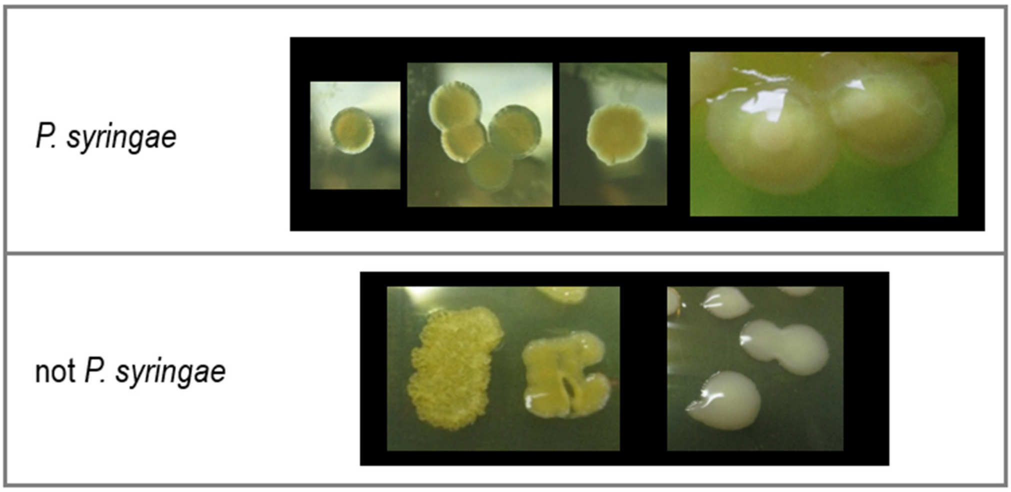

Supplement: Supplementary file 1 [file pathogens-11-00357-s001.zip › Morris_MDPI_IcelandicPs_Suppl_Fig_S1_definitive_12Mar22.pdf]
